# Advocating for the Fogarty International Center: An Unsung Hero for Global Health Research and Development

**DOI:** 10.4269/ajtmh.17-0611

**Published:** 2017-09-07

**Authors:** Jamie Bay Nishi

**Affiliations:** Director, Global Health Technologies Coalition

Before this year, the Fogarty International Center at the National Institutes of Health (NIH) received little air time on Capitol Hill. Though Fogarty has long been a “best buy” in our government—delivering significant scientific returns for global and American health while costing less than one quarter of 1% of the total NIH budget—it largely floated under the radar.

That was until May, when President Trump proposed eliminating the small but mighty Fogarty International Center in his fiscal year 2018 budget blueprint, *A New Foundation for American Greatness*. With this proposal, Members of Congress were faced with a big “what if.” What if Fogarty was eliminated from the NIH? What would that mean for global health, and what would that mean for American health?

This is a question that the Global Health Technologies Coalition (GHTC) was ready to answer. Our coalition of 27 nonprofit organizations^1^ working in global health research and development (R&D) has long championed the U.S. government’s role in global health R&D and highlighted the Fogarty International Center’s important role in accelerating science, partnerships, and technical assistance to advance new technologies for some of the world’s most pressing health challenges. Since its founding in 1968, Fogarty has played a pivotal role in global health research, driving important medical breakthroughs and vital international partnerships that benefit global and American health.

For example, scientists trained through Fogarty’s AIDS International Training and Research Program facilitated groundbreaking multi-country clinical trials that found that early administration of antiretroviral drugs had a significant and beneficial effect in preventing transmission of HIV to uninfected partners. This finding has revolutionized HIV/AIDS treatment and prevention around the world and at home in the United States, getting people on treatment sooner and saving millions of lives. In the aftermath of this discovery, the World Health Organization estimated in 2013 that early antiretroviral therapy could avert an additional 3 million deaths and prevent 3.5 million more new HIV infections by 2025.

Fogarty’s role in this breakthrough was critical, because Fogarty forged the international partnerships that made the underlying network of clinical trials possible. And since discoveries related to emerging or neglected infectious diseases can often only be made in low-resource settings where the disease burden is high, these types of clinical breakthroughs would not have been possible conducting research in the United States alone. It’s a powerful example of how engaging internationally and thinking globally yields dramatic returns for medical research and health outcomes around the world.

There are also more recent examples in which Fogarty-trained researchers have made critical contributions to global public health challenges. When the Ebola virus struck Guinea, Liberia, and Sierra Leone in 2014, disease investigators in Mali and Nigeria—who were trained in effective surveillance, isolation, and contact tracing techniques through Fogarty-funded programs—were essential to stopping disease transmission in those countries and mitigating a continent-wide pandemic. When Zika hit Latin America, Fogarty-trained scientists in Brazil, who were studying the links between Chagas disease and neurological disorders, quickly adapted their models to study the links between Zika and microcephaly. They also initiated work to identify the mechanisms through which Zika causes brain damage to better target research into effective treatment and prevention technologies.

To some these contributions may seem minor in the broader scope of the response to these outbreaks, but the global health R&D community understood how vital the actions of Fogarty-supported scientists were. Not only did they keep Americans (and the world) safe and healthy, they also provided the necessary first steps in advancing urgently-needed surveillance, detection, prevention, and treatment technologies for two under-resourced, under-researched diseases.

When he testified before Congress, Director of the National Institute for Allergy and Infectious Diseases Dr. Anthony Fauci shared some of these examples and called Fogarty “truly integral to all we do, both directly and indirectly, and internationally and domestically.” He answered the question of why the Fogarty International Center—and global health R&D—matters, and he described the important gains the medical research community makes through working globally.

His testimony, and the stories shared by Fogarty advocates like GHTC, are compelling because they underscore the big “what if” proposed in the President’s budget: What if the Fogarty International Center didn’t exist? What if Fogarty researchers hadn’t contained the Ebola outbreak to Sierra Leone, Guinea, and Liberia? What if the United States hadn’t helped train scientists in Brazil to understand the links between vector-borne viruses and brain damage? What if Fogarty hadn’t advanced revolutionary research in HIV/AIDS prevention? What would this mean for Americans? What would this mean for the world?

And indeed, global and American health will suffer a significant setback without the Fogarty International Center—a center with a modest budget of $72 million in the context of $3.8 trillion in annual U.S. federal spending. In our increasingly interconnected world, when the phrase “diseases know no borders” has almost become trite, the value proposition of the Fogarty International Center has never been more clear. Thanks to strong advocacy from the global health R&D community, Members of Congress are starting to pay attention, they are understanding Fogarty’s vital and unique role, and they are becoming champions of Fogarty themselves.

This newfound political support is exemplified by the remarks of Representative Tom Cole, Chairman of the U.S. House Appropriations Subcommittee on Labor, Health and Human Services, who said in defending the Fogarty International Center and other U.S. investments in global health research, “Do you want to deal with Ebola in West Africa or do you want to deal with it in West Dallas? ...The federal government defending you from Ebola is probably as important as defending you from a terrorist attack because a pandemic will kill more people than a terrorist attack will.”

This is reason to celebrate, and reason to continue to tell stories of the remarkable accomplishments achieved through the Fogarty International Center. Together we can ensure Fogarty’s important work is recognized, is sustained, and helps to advance health for future generations.

## Footnotes

^1^ The Global Health Technologies Coalition is coalition of more than 25 nonprofit organizations advancing policies to accelerate the creation of new drugs, vaccines, diagnostics, and other health tools that bring healthy lives within reach for all people. The ASTMH is a member organization.

